# Early Initiation of Tocilizumab in Clinically Isolated Aortitis

**DOI:** 10.7759/cureus.4479

**Published:** 2019-04-16

**Authors:** Irvin J Huang, Trevor Pugh, Jean Liew

**Affiliations:** 1 Internal Medicine, University of Washington, Boise, USA; 2 Rheumatology, University of Washington, Seattle, USA

**Keywords:** tocilizumab, vasculitis, aortitis, steroids, glucocorticoids

## Abstract

Clinically isolated aortitis can arise from infectious or inflammatory etiologies. Glucocorticoids are the first-line therapy for inflammatory causes of aortitis such as large-vessel vasculitis. However, prolonged steroids use is associated with numerous side effects. We present a case of a 60-year-old woman with clinically isolated aortitis who received early treatment with tocilizumab to avoid prolonged steroid use.

## Introduction

Aortitis can arise from infectious or inflammatory etiologies. Corticosteroids are the mainstay therapy in inflammatory conditions such as large vessel vasculitis [1-2]. We present a case of clinically isolated aortitis due to large vessel vasculitis, which was treated with the early initiation of tocilizumab and allowed for a rapid glucocorticoid taper.

## Case presentation

A 60-year-old woman with a past medical history of remote breast cancer and depression was evaluated at an outside hospital emergency department for high fevers, myalgia, fatigue, productive cough, and chills. She had a recent gastrointestinal illness after eating fish and salad at a new restaurant, which spontaneously resolved. Two days afterward, she began experiencing fevers associated with fatigue and drenching sweats that occurred twice daily and were not alleviated with antipyretics. Additionally, she reported headaches, sinus congestion, and a sore throat that resolved with antibiotics prescribed for sinusitis.

**Figure 1:**
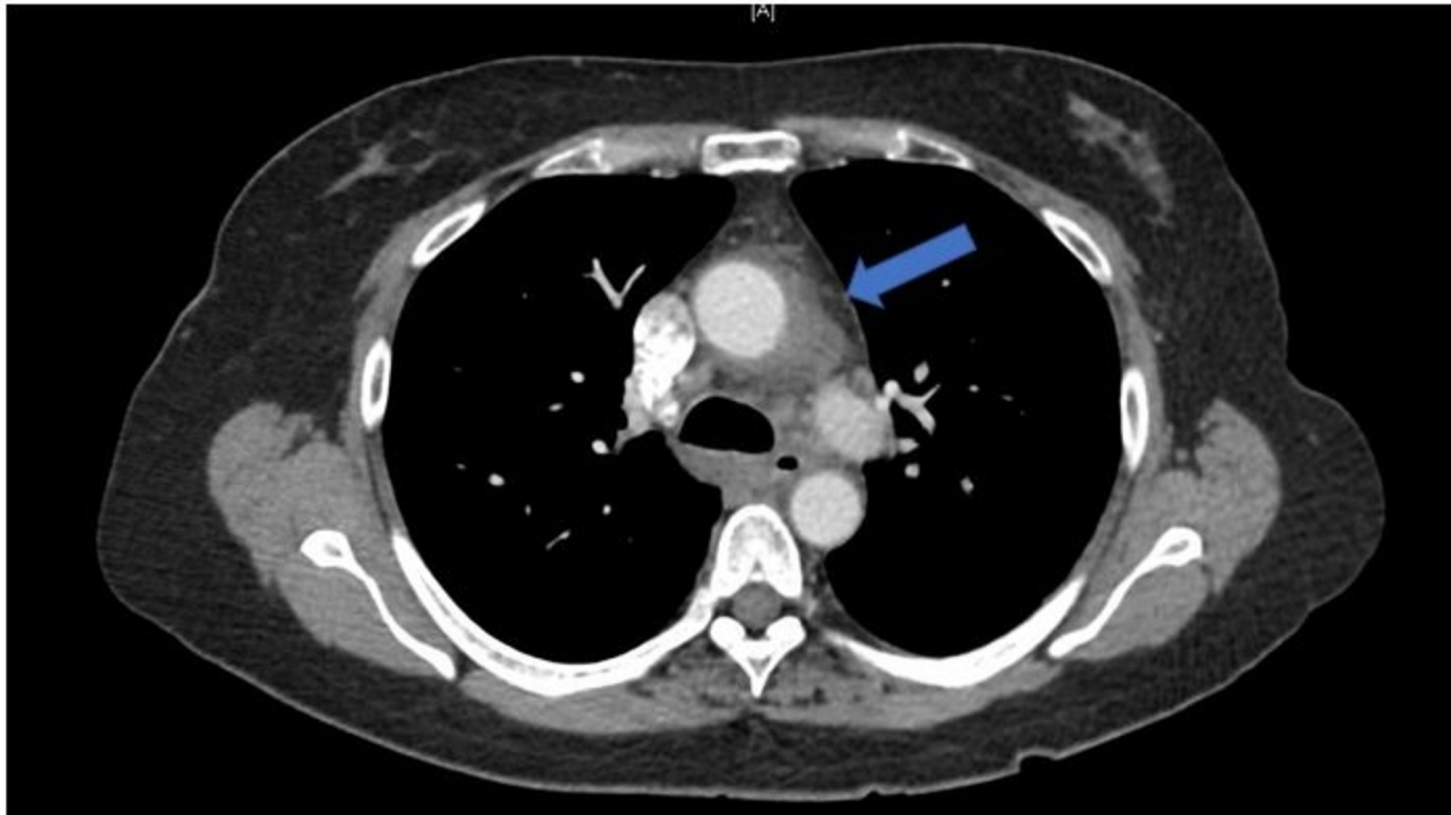
Aortitis on CT chest Computed tomography (CT) chest without contrast obtained during the March 2018 admission for fevers and constitutional symptoms. Diffuse fat stranding is seen surrounding the ascending aorta and aortic arch (arrow), consistent with aortitis.

Her vital signs were significant for a temperature of 39.4°C. Her physical exam did not disclose temporal tenderness, asymmetrical pulses, conjunctival injection, oral ulcers, cervical lymphadenopathy, or rashes. Her initial laboratory studies were remarkable for a white blood cell count (WBC) of 21,700/uL, C-reactive protein (CRP) of 26 mg/L, and erythrocyte sedimentation rate (ESR) of 74 mm/hr. Computed tomography (CT) of the chest with contrast showed diffuse, abnormal soft tissue around the ascending aorta and aortic arch with fat stranding, which was consistent with aortitis (Figure 1). She was transferred to our hospital in Seattle, Washington, for rheumatological management of aortitis.

On repeat laboratory evaluation, WBC was 18,000/uL, ESR was 105 mm/hr, and CRP was 319.7 mg/L. Rheumatoid factor, anti-cyclic citrullinated peptide, antinuclear antibody, anti-double-stranded DNA, antineutrophil cytoplasmic antibody, and serum IgG4 subclasses were unrevealing. Her persistent double-quotidian fevers, elevated inflammatory markers, and aortic findings on CT were concerning for an infectious versus inflammatory aortitis. Potential infectious etiologies of aortitis included tuberculosis (TB), human immunodeficiency virus (HIV), syphilis, and enteric pathogens such as Salmonella. Serologic and fecal testing for these infectious etiologies were negative.

The differential for noninfectious, inflammatory conditions included giant cell arteritis (GCA), Takayasu’s arteritis, anti-neutrophilic cytoplasmic autoantibody (ANCA) vasculitis, systemic lupus erythematosus (SLE), rheumatoid arthritis (RA), immunoglobulin G4 (IgG4)-related disease, and Erdheim-Chester disease. Her lack of suggestive clinical history and negative serologies made RA, SLE, and ANCA vasculitis unlikely. Her high fevers and significantly elevated inflammatory markers were less suggestive of IgG4-related disease or Erdheim-Chester disease, and imaging did not demonstrate a suggestive pattern of involvement in other organs. There was also no evidence of malignancy on axial imaging.

**Figure 2:**
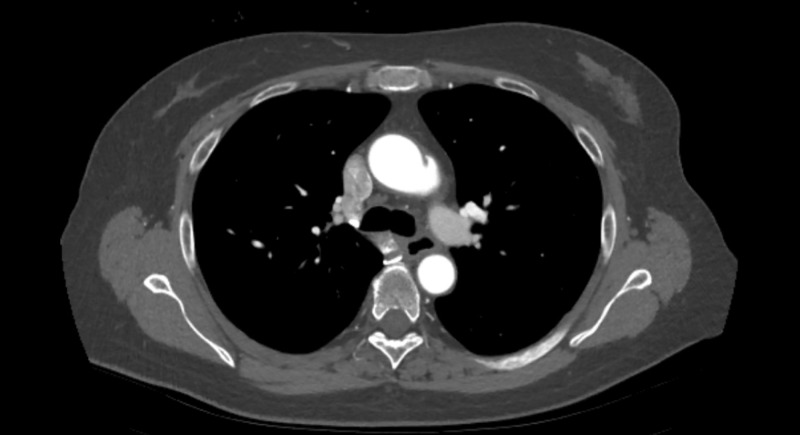
Follow-up CT chest Follow-up computed tomography (CT) chest demonstrating the attenuation of inflammation. The pseudoaneurysms were believed to be sequelae of the initial episode. They have been stable on further repeat imaging.

The patient was diagnosed with isolated aortitis given her presenting constitutional symptoms, significantly elevated inflammatory markers, and CT chest finding of diffuse fat stranding surrounding the ascending aorta and aortic arch. Her isolated aortitis may be a limited variant of GCA or Takayasu’s arteritis although she did not have the classical symptoms of either [3]. Ultrasound of the temporal arteries and vessels of the upper extremities did not demonstrate a characteristic “halo sign,” and temporal artery biopsy was deferred given the lack of suggestive clinical features of GCA. The patient was initiated on prednisone therapy at 60 mg daily with a taper over four months. However, due to steroid intolerance, including fatigue and poor sleep, she was started on weekly tocilizumab 162 mg subcutaneous injections within one month of diagnosis. Her elevated inflammatory markers and symptoms resolved quickly and she remains in remission at 10 months of follow-up. Follow-up CT chest demonstrated the attenuation of inflammation around the aorta (Figure 2). Even though pseudoaneurysms were noted, these were felt to be likely sequelae of the initial episode. They were stable on further repeat imaging. We plan to continue with tocilizumab for at least 18 months, although the optimal duration of therapy is unclear [4].

## Discussion

Clinically isolated aortitis can be a manifestation of either infectious or non-infectious inflammatory etiologies [5]. Inflammatory causes include GCA, Takayasu’s arteritis, and IgG4-related disease more commonly and SLE, RA, Cogan’s syndrome, Behçet’s disease, relapsing polychondritis, and ankylosing spondylitis more rarely. According to the 2012 Chapel Hill Consensus Conference, isolated aortitis is classified as a single organ vasculitis, although they may just be limited variants of GCA or Takayasu’s arteritis [3]. Glucocorticoids are the first-line therapy for large-vessel vasculitis, although their use is associated with potential side effects that increase with higher cumulative doses. Tocilizumab, an interleukin 6 (IL-6) receptor monoclonal antibody, was approved for the treatment of GCA after a randomized trial found significantly higher rates of sustained remission in the tocilizumab-treated group as compared to the group receiving glucocorticoids alone [4]. In case series and case reports of aortitis secondary to underlying GCA, Takayasu’s arteritis, ANCA vasculitis, and relapsing polychondritis, tocilizumab has been used successfully (Table 1) [6-13]. This significant response to tocilizumab suggests that the IL-6 cytokine is involved as a central component in the development of aortitis independent of the concurrent disease syndrome. Additionally, the patients’ improvement on tocilizumab in our literature review appears to be unrelated to previous therapies administered. In our case, tocilizumab was started early in the course of disease with a rapid and sustained response.

**Table 1:** Case series of tocilizumab for the treatment of aortitis as the primary presentation of giant cell arteritis, Takayasu’s arteritis, polymyalgia rheumatica, relapsing polychondritis, ANCA vasculitis, or retroperitoneal fibrosis Abbreviations: MTX: Methotrexate; CYC: Cyclophosphamide; MMF: Mycophenolate mofetil; ETN: Etanercept; IFX: Infliximab; ADA: Adalimumab; CyA: Cyclosporine A; LFN: Leflunomide; SSZ: sulfasalazine; ANCA: Anti-neutrophilic cytoplasmic autoantibody

Citation	Age	Gender	Underlying condition	Tocilizumab therapy regimen	Outcome
Narshi C. et al. [11]	43	Female	Relapsing polychondritis with collapse of the nasal bridge, recurrent ocular inflammation, bilateral auricular and costochondritis followed by aortitis. Previously on corticosteroids and IFX, followed by ADA which was increased to 10 mg/kg with MTX and deflazacort	8 mg/kg in combination with deflazacort	18 month follow-up with down-titration of deflazacort to 6 mg daily
Kawai, M. et al. [10}	29	Female	Relapsing polychondritis with auricular, nasal and costochondral pain with thickening of laryngotracheal walls with severe airway narrowing and bronchial wall thickening. Previously on CyA, tacrolimus, CYC and IFX 3 mg/kg	8 mg/kg in combination with prednisone 40 mg	9 month follow-up with decrease in prednisone to 15 mg and 10 mg in one year
Kawai, M. et al. [10]	52	Male	Relapsing polychondritis with airway narrowing and airway thickening. Previously on MTX 7.5-10 mg/week and prednisolone 15-30 mg/day	8 mg/kg in combination with prednisolone 15 mg	5 months follow-up, prednisolone decreased to 10 mg
Stael, R. et al. [9]	25	Male	Relapsing polychondritis with asymmetrical oligoarthritis, scleritis and endocarditis. Previously on SSZ 3 g daily and piroxicam 20 mg, transitioned to ETN and developed endocarditis; then IFX and MTX 15 mg with prednisolone 20mg.	8mg/kg in combination with prednisolone 10 mg and MTX 15 mg weekly	5 month follow-up, developed SVT, markedly improved symptoms in one year
Ashraf, F.A.M. et al. [12]	62	Male	Polymyalgia rheumatica with fatigue, night sweats, aortic valve insufficiency, aortitis with aortic aneurysm. Previously on methylprednisolone	Unspecified	Inflammatory markers continued to be normal at time of publication
Elourimi, G. et al. [8]	59	Female	Relapsing polychondritis with nasal, bilateral auricular chondritis with pericarditis, episcleritis. Previously on prednisone 1 mg/kg daily, gradually tapered to 5 mg/day, episcleritis recurred.	8mg/kg with prednisone 70 mg	3 month follow-up with prednisone decreased to 5 mg
Loricera, J. et al. [6]	7	Female	Takayasu’s arteritis. Previously on MTX, CYC, MMF, ETN, IFX, Prednisone 30 mg	8mg/kg with prednisone 30 mg	24 month follow-up, prednisone decreased to 0 mg
Loricera, J. et al. [6]	57	Female	Takayasu’s arteritis. Previously on CYC	8mg/kg with prednisone 45 mg	18 month follow-up, prednisone decreased to 5 mg
Loricera, J. et al. [6]	26	Female	Takayasu’s arteritis. Previously on MTX, AZA, IFX	8mg/kg with prednisone 50 mg	12 month follow-up, prednisone decreased to 7.5 mg
Loricera, J. et al. [6]	16	Female	Takayasu’s arteritis. Previously on MTX, ADA	8mg/kg with prednisone 50 mg	12 month follow-up, prednisone decreased to 7.5 mg
Loricera, J. et al. [6]	45	Female	Takayasu’s arteritis. Previously on MTX, AZA, MMF, IFX	8 mg/kg with prednisone 25 mg	13 month follow-up, prednisone decreased to 0 mg
Loricera, J. et al. [6]	41	Female	Takayasu’s arteritis. Previously on MTX, ADA, IFX	8mg/kg with prednisone 40 mg	3 month follow-up, prednisone decreased to 10 mg
Loricera, J. et al. [6]	46	Female	Takayasu’s arteritis. Previously on MTX	8mg/kg with prednisone 25 mg	4 month follow-up, prednisone decreased to 5 mg
Loricera, J. et al. [6]	77	Female	Giant cell arteritis. Previously on MTX	8mg/kg with prednisone 10 mg	5 month follow-up, prednisone decreased to 2.5 mg
Loricera, J. et al. [6]	59	Female	Giant cell arteritis. Previously on MTX	8mg/kg with prednisone 60 mg	16 month follow-up, prednisone decreased to 5 mg
Loricera, J. et al. [6]	65	Female	Giant cell arteritis. Previously on MTX	8mg/kg with prednisone 17.5 mg	20 month follow-up, prednisone decreased to 0 mg
Loricera, J. et al. [6]	67	Female	Giant cell arteritis. Previously on MTX	8mg/kg with prednisone 10mg	6 month follow-up, prednisone decreased to 0 mg
Loricera, J. et al. [6]	74	Female	Giant cell arteritis. Previously on MTX	8mg/kg	11 month follow-up, inflammatory markers undetectable
Loricera, J. et al. [6]	64	Female	Giant cell arteritis. Previously on MTX	8mg/kg with prednisone 15 mg	3 month follow-up, prednisone decreased to 10 mg
Loricera, J. et al. [6]	53	Male	Giant cell arteritis. Previously on MTX	8mg/kg with prednisone 30 mg	5 month follow-up, prednisone decreased to 10 mg
Loricera, J. et al. [6]	50	Female	Relapsing Polychondritis with aortitis. Previously on MTX, CyA, LFN, CYC, IFX	8mg/kg with prednisone 30 mg	20 month follow-up, prednisone decreased to 5 mg
Loricera, J. et al. [6]	75	Male	Retroperitoneal fibrosis with aortitis. No previous treatment.	8mg/kg	17 month follow-up.
Takenaka, K. et al. [7]	47	Female	ANCA associated vasculitis with hypertrophic pachymeningitis and aortitis. Previously on CYC and prednisolone 40 mg, increased to 50 mg.	400 mg/month with prednisolone 50 mg	1 year follow-up, prednisolone decreased to 4 mg

## Conclusions

Inflammatory aortitis may be the sole presenting manifestation of a large vessel vasculitis. Although steroids are the first-line therapy for a variety of inflammatory conditions that may cause aortitis, their prolonged use is associated with a myriad of side effects. We advocate for the early consideration of tocilizumab for the management of inflammatory aortitis, a potentially life-threatening condition.
